# The Experiences of Student Nurses With Dyslexia in Clinical Practice in the United Kingdom: A Literature Review

**DOI:** 10.1111/jan.16900

**Published:** 2025-03-25

**Authors:** Rachel Burton, Obrey Alexis

**Affiliations:** ^1^ Registered Nurse Oxford Brookes University Swindon UK; ^2^ Faculty of Health and Life Sciences Oxford Brookes University Swindon UK

**Keywords:** clinical, dyslexia, education, experiences, nursing, practice, students, supervision, support

## Abstract

**Aim:**

To explore the experiences of student nurses with dyslexia in clinical practice in the United Kingdom.

**Design:**

A systematic literature review of qualitative research.

**Methods:**

Three databases—CINAHL, MEDLINE and the British Education Index, were searched for original articles, using keywords to find peer‐reviewed papers published between 1995 and 2024. Two reviewers independently evaluated the studies for inclusion, and the selected studies were critically appraised using the CASP tools. The extracted data were thematically analysed and synthesised.

**Results:**

Six studies were identified. Thematic analysis revealed four themes: Disclosing dyslexia, patient safety, compensatory coping strategies and support from practice assessors/supervisors.

**Conclusions:**

Student nurses with dyslexia have a variety of difficulties in clinical practice that call for continual assistance from their practice supervisors and assessors, including ward managers. Students must reveal their dyslexia to receive support, which can be a difficult and complicated process. Students should capitalise on their skills and employ compensating coping mechanisms to maintain patient safety. Further research is required to explore appropriate adjustments and the current level of help needed to support student nurses with dyslexia.

**No Patient or Public Involvement:**

This study did not include patient or public involvement in its design, conduct or reporting.

**Summary**

- Implications and recommendations for clinical practice
  ○ Practice assessors should collaborate with student nurses to identify reasonable adjustments to accommodate dyslexia and support learning outcomes.
  ○ Regular check‐ins by practice assessors and supervisors can help guide students in navigating clinical placements effectively and adapting to their learning needs.
  ○ Placement coordinators should work with students with dyslexia to identify suitable placement areas, potentially starting with quieter placements to build confidence and skills.
  ○ Implement a Dyslexic Buddy Scheme to allow students to share experiences and coping strategies.
  ○ Provide training for practice assessors and supervisors on learning difficulties, reasonable adjustments and student support.
  ○ Educate student nurses about dyslexia and other learning difficulties to foster a more inclusive environment for future colleagues and students.

## 1 Introduction

Dyslexia is a set of processing challenges that affect reading and spelling development (British Dyslexia Association 2023). Individuals with dyslexia often achieve lower literacy levels than expected based on their age and education (Myhill 2022). Key indicators of dyslexia across languages and age groups include difficulties in reading and spelling fluency. Various genetic and environmental factors influence the nature and progression of dyslexia. It exists on a spectrum, varying in severity, and can also impact skills such as math and language acquisition (British Dyslexia Association 2023). A common challenge associated with dyslexia is phonological processing, but other factors like working memory and processing speed also contribute to its effects. Dyslexia often co‐occurs with other developmental issues, including language disorders, dyscalculia, ADHD and coordination problems (The SpLD Assessment Standards Committee (SASC) et al. 2024; British Dyslexia Association 2023).

Dyslexia is a significant aspect of neurodiversity, affecting a considerable portion of the population, estimated to be between 10% and 20% (Myhill 2022). In the United Kingdom, approximately one in ten people is believed to have dyslexia, with over 3.3 million adults in the workplace experiencing some form of dyslexia (British Dyslexia Association 2023). Furthermore, Jelly (2014) suggests that around three to 10% of nurses admit to being dyslexic. However, there is a nature of selective disclosure (Glazzard and Stones 2021).

In the UK, the training of nurses has undergone a number of significant developments over the past three decades. Recently, nursing students undertake a 3‐year programme based at university, and they split their time equally between university‐based theoretical modules and hospital‐based clinical placements, which must consist of 50% theory (2300 h) and 50% practice (2300 h) (Nursing and Midwifery Council (NMC) 2010). A practice assessor is a registered health professional who assesses students' practice learning. They work with students to help them learn and provide feedback on their progress in clinical practice. Unlike a practice supervisor, who is a registered health and social care professional who helps students learn and develop. They work with students in practice placements, providing feedback and evidence of students' progress to the practice assessor. These practice assessors and practice supervisors will work with students with dyslexia to enable them to achieve proficiency, culminating in registration with the Nursing and Midwifery Council in the UK.

Conducting a literature review on the experiences of student nurses with dyslexia in clinical practice in the UK is crucial for understanding the challenges they face. Additionally, providing adequate support to student nurses with dyslexia can enhance patient care and help them become confident and competent registered nurses. The rationale for this literature review is to explore the experiences of student nurses with dyslexia in clinical practice in the UK.

This review will focus solely on student nurses in the UK because the educational system, support mechanisms, and clinical placement frameworks may significantly differ from those in other countries. Understanding these unique challenges within the UK context will yield more relevant insights and recommendations for improving support for student nurses with dyslexia in the UK.

Dyslexia is a globally recognised condition that impacts individuals across various educational fields, including nursing (International Dyslexia Association 2022). The support systems available for student nurses with dyslexia differ from one country to another, reflecting a range of educational frameworks and policies (Elliott and Grigorenko 2024). Many nations are actively working to improve their healthcare systems and educational structures, making it essential to recognise findings from the UK. These insights can guide policymakers and educators worldwide in their efforts to enhance the quality of nursing education and practice by providing valuable comparisons and fostering cross‐cultural insights.

Moreover, considering that patient safety is a universal priority within healthcare (World Health Organisation 2023), understanding how student nurses with dyslexia perceive and manage patient safety in the UK can contribute significantly to global discussions aimed at improving patient care and safety protocols in clinical settings.

## 2 Aim

The aim of this systematic literature review of qualitative research is to explore the experiences of student nurses with dyslexia in clinical practice in the UK.

## 3 Methods

The literature review seeks to explore the experiences of student nurses with dyslexia in clinical practice within the UK. Qualitative research is particularly well suited for investigating these experiences, as it offers a deep and nuanced understanding of their personal challenges, practices and coping strategies. Consequently, qualitative research papers are deemed the most appropriate for addressing this inquiry.

### 3.1 Design

A literature review will be conducted to identify and summarise any empirical data that meets the pre‐specified inclusion and exclusion criteria within the scope of the research question (Snyder 2019).

### 3.2 Search Strategy

Original primary qualitative research articles were sought from three databases: CINAHL, MEDLINE and the British Education Index. According to Bramer et al. (2017), conducting a systematic review requires scanning numerous databases in order to be efficient and adequate. Using several databases for qualitative research ensures a thorough search and identification of relevant material (Aveyard 2023).

The following keywords were used across all databases: (experiences OR attitudes OR views OR feelings) AND (student nurse OR nursing student OR nursing students OR undergraduate nurse) AND (dyslexia or dyslexic or neurodiversity or neurodiverse).

**FIGURE 1:**
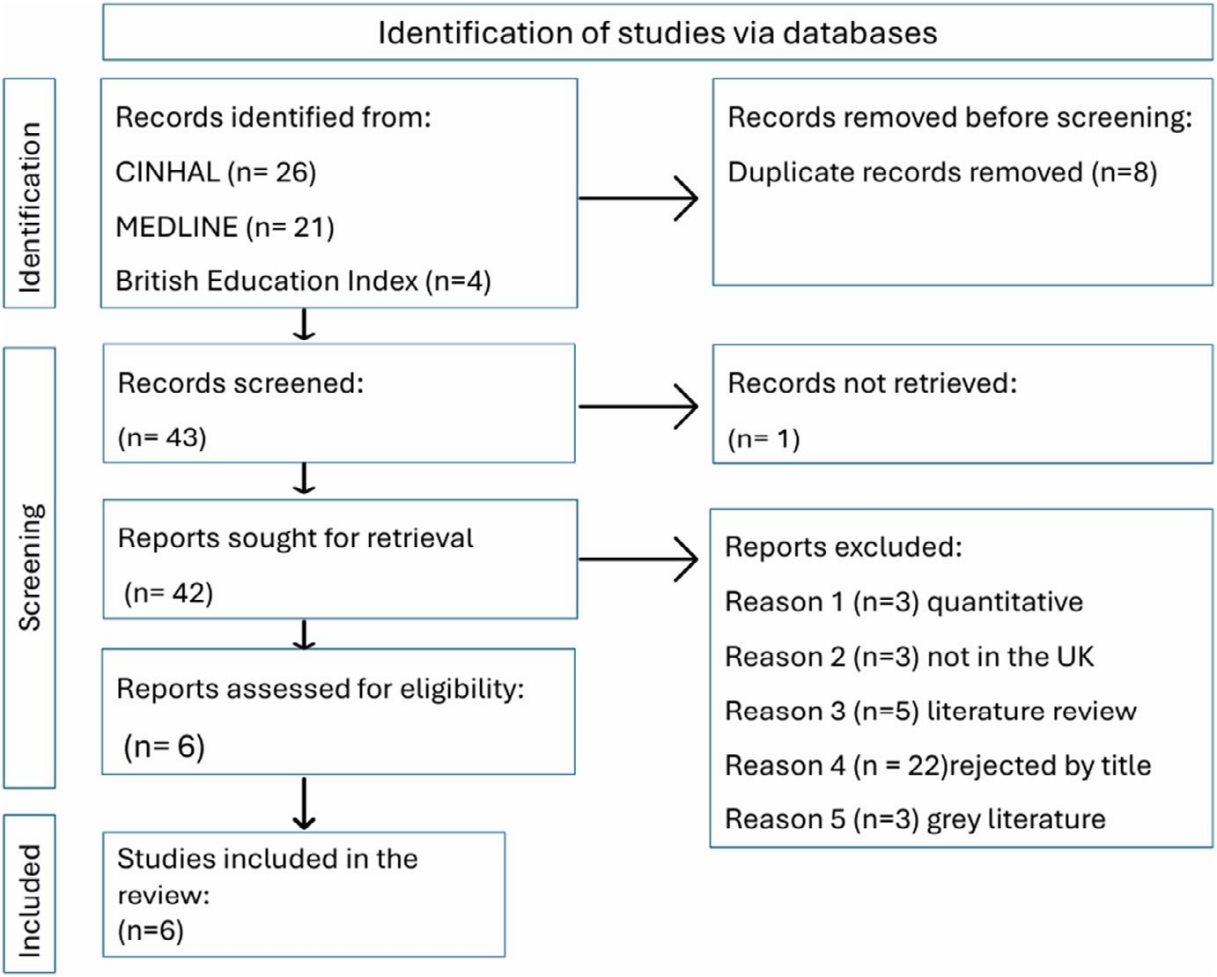
PRISMA 2020 diagram (Page et al. 2021).

The review of qualitative research covered the period from 1995 to 2024. The exploration of literature from post‐1995 regarding nurses with dyslexia in clinical practice is essential, particularly in light of the significant implications of the Equality Act 2010. This legislation superseded the Disability Discrimination Act 1995, providing more robust protections and setting forth requirements for reasonable adjustments. By concentrating on literature from this period, the research ensures a focus on the most pertinent and current context, accurately reflecting the legal frameworks and practices that shape the support, accommodations, and experiences of nurses with dyslexia in the clinical setting. The results can be seen in the prisma diagram in Figure 1.

### 3.3 Inclusion and Exclusion Criteria

Inclusion criteria:

- Student nurses with dyslexia.
- Publications from the UK.
- English language only.
- Studies employing qualitative research designs.
- Full‐text availability.
- Studies published from 1995 to 2024.

Exclusion criteria

- Nurses or other healthcare professionals with dyslexia.
- Publications from outside the UK.
- Studies using quantitative research designs, literature reviews, grey literature and non‐English language papers.
- Review articles, meta‐syntheses or meta‐analyses.
- Studies published before 1995.

### 3.4 Quality Appraisal

The Critical Appraisal Skills Programme (CASP) tool checklists for qualitative research in assessing their efficacy, reliability and validity were applied to the chosen literature to assess their quality. The CASP tool also aids in identifying potential biases in the design and execution of research papers (Aveyard 2023). Nadelson and Nadelson (2014) highlight that the CASP tool is widely used in nursing research for its concise and compelling nature. Consequently, the six papers were deemed suitable and valid for inclusion in the literature review following the CASP tool application. The characteristics of the studies can be found in Table 1.

**TABLE 1:** Characteristics of included studies.

Paper number	Author, year, UK location	Title	Aim	Research design	Sample size	Data collection methods	Outcomes
1	Morris and Turnbull 2006, England	Clinical experiences of students with dyslexia	To explore the experiences that dyslexia student nurses have in clinical practice and its potential impact	Qualitative study	18 student nurses with a formal diagnosis of dyslexia	Semi‐structured interviews	Five themes emerged the following: the need for more time, self‐managing strategies, choice of future work settings, disclosure and emotional aspects
2	Morris and Turnbull 2007, England	The disclosure of dyslexia in clinical practice: Experiences of student nurses in the United Kingdom	To explore student nurses' experiences with dyslexia when and if disclosing their disability in clinical practice	Qualitative study	18 student nurses with a formal diagnosis of dyslexia	Semi‐structured interviews	Most participants felt disclosing their disability in clinical practice was threatening and stressful and felt that a level of maturity and confidence was required. Longer placements resulted in more students disclosing their dyslexia
3	Ridley 2011, England	The experiences of nursing students with dyslexia	To explore one university's experiences with dyslexia among pre‐registration nursing students	Qualitative study	7 student nurses with a formal diagnosis of dyslexia	Semi‐structured interviews	Participants felt they had a duty to disclose dyslexia to safeguard those in their care but feared discrimination and ridicule. Participants were self‐aware, and not all felt their dyslexia was a disability
4	Child and Langford 2011, England	Exploring the learning experiences of nursing students with dyslexia	Understanding the experiences student nurses have when learning in clinical practice to identify support improvements	Qualitative study	12 student nurses, 6 with dyslexia and 6 without dyslexia	Semi‐structured interviews	Three themes that emerged were the need for advocacy, the need for mentorship and the value of worked‐based learning days
5	Crouch 2019, England	Perceptions of the possible impact of dyslexia on nursing and midwifery students and of the coping strategies they develop and/or use to help them cope in clinical practice	To explore the impact that dyslexia has on student midwives and nurses and what coping strategies are used to cope in clinical practice	Qualitative study	12 nursing and midwifery students and 22 mentors	Semi‐structured interviews	Three core themes emerged: strategies used to manage the perceived impact of dyslexia, very good and helpful coping strategies and perceptions of the impact of dyslexia on student practise
6	King 2019, England	Exploring student nurses' and their link lecturers experience of reasonable Adjustments in clinical practice	To explore student nurses' experiences with requiring reasonable adjustments and their link lectures associated with providing support in clinical practice	Qualitative study	7 student nurses and 3 link lectures	Semi‐structured interviews	Three themes highlighted were being professional, defining reasonable adjustments and supporting students

## 4 Results

In this section, the characteristics of each paper will be identified, and a thematic analysis of the research papers will be used to ascertain emergent themes.

### 4.1 Characteristics of the Included Studies

All six studies included are qualitative research papers (Morris and Turnbull 2006, Morris and Turnbull 2007; Ridley 2011; Child and Langford 2011; Crouch 2019; and King 2019) and were published in the UK. They vary in aims, publication dates, sample sizes and data collection methods, all provided in an overview of the characteristics of the included studies in Table 1.

### 4.2 Year of Publication

The research papers chosen for the review were published between 2006 and 2019. The introduction of the Equality Act 2010 may be the reason for a rise in research in this area. The Equality Act 2010 raised greater awareness and legal protection, and research focused on dyslexia as a protected characteristic aimed to improve support for people with dyslexia in various contexts (Pyper et al. 2023).

### 4.3 Country of Origin

Although the inclusion criteria for this review stipulated that research should be conducted in the UK, all papers were from England. No papers were identified from the other three home nations: Wales, Scotland, or Northern Ireland.

### 4.4 Aim and Research Design

All of the research takes an exploratory and descriptive approach to the perspectives or views of student nurses with dyslexia. All six articles included in the review are qualitative in nature.

### 4.5 Sample

In total, throughout the six studies, 68 students with dyslexia were included across six Universities, all of which received a diagnosis of dyslexia before undertaking their nursing course and participating in the research. (Morris and Turnbull 2006, 2007; Crouch 2019; King 2019; Child and Langford 2011; Ridley 2011).

None of the papers disclosed the ethnicity, nationality or cultural backgrounds of the participants (Morris and Turnbull 2006, 2007; Crouch 2019; King 2019; Child and Langford 2011; and Ridley 2011).

### 4.6 Data Collection and Analysis Methods

All six research studies employed a purposeful sampling strategy by sending a letter of invitation to potential participants. The letters were sent to all nursing students at the identified Higher Education Institutions. Five of the research studies sent letters stating that if the students had a diagnosis of dyslexia, they were then eligible to participate in the study and respond to the invitation (Morris and Turnbull 2006, 2007; Crouch 2019; King 2019; and Ridley 2011). In one study, students without dyslexia were invited to participate and were sent the same letter explaining that it was a qualitative comparison study (Child and Langford 2011).

All six of the studies obtained written consent from the participants before starting. They used face‐to‐face semi‐structured interviews, either video or voice‐recorded, and subsequently transcribed them. (Morris and Turnbull 2006, 2007; Crouch 2019; King 2019; Child and Langford 2011; and Ridley 2011). The data analysis varied in the process and method, but most of the studies employed a thematic analysis, identifying, coding and reporting patterns within the research data (Braun and Clarke 2019). Crouch (2019) used grounded theory to attempt to bridge a gap between research and theory, which assists in gathering rich data (Glaser and Strauss 1967).

### 4.7 Data Synthesis for This Review

**Diagram 1:**
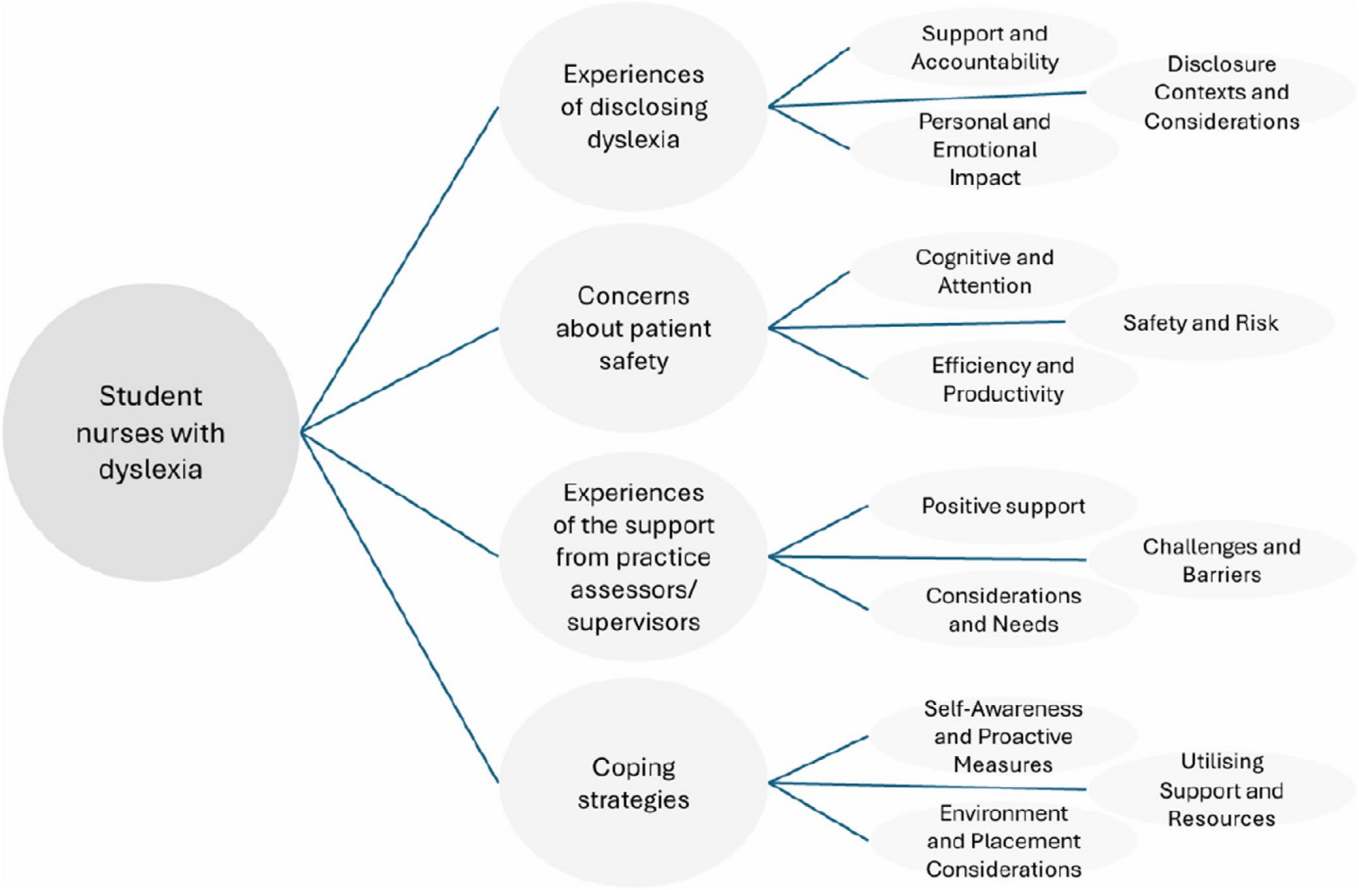
Thematic findings.

The synthesis process consists of summarising, collating and analysing similarities or differences between findings from research papers (Braun and Clarke 2019). The extracted data from the six research papers were thematically analysed, and the findings emerged into four themes: disclosure of dyslexia, patient safety, support from practice assessors/supervisors and compensatory coping strategies (Diagram 1).

### 4.8 Disclosing Dyslexia

Disclosure of dyslexia is crucial for student nurses to access reasonable adjustments under the Equality Act 2010. However, most student nurses with dyslexia prefer to disclose their condition to university educators, as they anticipate better support from educational institutions. This preference indicates a robust support system and accountability within educational settings, making it easier for students to seek the necessary accommodations.

In contrast, many student nurses are less inclined to disclose their disability during clinical placements due to stigma and potential discrimination (Morris and Turnbull 2007; Ridley 2011; Child and Langford 2011; King 2019). This situation highlights the personal and emotional impact of dyslexia disclosure in different contexts, as the fear of negative reactions can deter students from seeking the support they need. Nevertheless, those who choose to disclose in clinical practice often do so to ensure patient safety (Morris and Turnbull 2007; Ridley 2011; Crouch 2019), underscoring the sense of responsibility and accountability that drives their decision. Student nurses with dyslexia actively decide whether to disclose their disability during clinical placements (Morris and Turnbull 2006, 2007; Crouch 2019; King 2019; Child and Langford 2011; Ridley 2011). Their decisions reflect their personal experiences and emotional resilience in managing their condition.

Morris and Turnbull (2007) investigated the clinical experiences of 18 student nurses diagnosed with dyslexia, focusing on their challenges and coping strategies. Twelve out of the 18 students chose to disclose their dyslexia in clinical practice, identifying it as a complex and stressful decision made to protect patient safety and seek support. Extended placements facilitated disclosure, whereas shorter placements allowed students to hide their difficulties by avoiding tasks that could highlight their dyslexia. Eight students felt that disclosing their dyslexia prompted extra support from practice assessors and supervisors, but only four felt their placements were adaptable to their needs.

Fear of stigma significantly affected students' willingness to disclose dyslexia (Ridley 2011). Furthermore, Child and Langford's (2011) study identified that all student participants disclosed their condition to their practice assessors, though many were hesitant due to negative past experiences, including discriminatory behaviour from assessors. Most students reported encountering discrimination or judgmental attitudes. The study highlighted the positive impact of universities informing placements about students with dyslexia beforehand, although one participant viewed this as a breach of confidentiality. Students found early disclosure to practice assessors helpful. Moreover, the findings from King (2019) indicate that while students understood reasonable adjustments in academic settings, they lacked knowledge about implementing them in clinical placements, which affected their perceived need to disclose their dyslexia.

Overall, the disclosure of dyslexia among student nurses is deeply influenced by the support and accountability provided in both educational and clinical settings, the personal and emotional impact of disclosure decisions and the specific contexts and considerations surrounding disclosure. Balancing the need for reasonable adjustments with the complexities of clinical practice remains a significant challenge.

### 4.9 Patient Safety

A key concern highlighted in the research papers is the awareness of patient safety risks among student nurses with dyslexia. Student nurses with dyslexia have identified perceived risks to patient safety that include medication calculations and errors, organisational skills, memory recall, communication and documentation. These factors underscore the need for greater awareness of dyslexia. By acknowledging these challenges, healthcare providers can implement reasonable adjustments and support systems to assist student nurses with dyslexia. Such measures may include providing assistive technologies, allowing additional time for tasks and assessments, and offering extra training and resources to enhance skills in challenging areas. Ultimately, the objective is to ensure that all student nurses, including those with dyslexia, can carry out their responsibilities safely and effectively, thereby upholding the high standards and safety of patient care.

In a grounded study conducted by Crouch (2019), the impact of clinical practice on 12 midwifery and nursing students was examined. Students reported concerns that their dyslexia could affect patient safety, particularly in medication administration. They expressed worries about correctly reading prescriptions from doctors, which were often unclear. Many students found drug calculations to be time‐consuming, complex and stressful, viewing them as risky. One participant admitted to making an error in documentation, stating, ‘I write in milligrams when it is really millilitres. Even though I have given the patient the right dose, it might appear that I have administered an incorrect dose.’ Another participant noted issues with writing numbers in the wrong sequence, such as 21 instead of 12, which poses a risk related to medication dosages.

In Ridleys' (2011) study, patient safety concerns could be linked to retaining information and being easily distracted. One participant disclosed being unable to concentrate in a noisy environment, resulting in not hearing the doctor. Self‐doubt and confusion could lead to patient safety concerns and students' confidence in raising concerns.

Documentation was another concern regarding patient safety. Students reported frequently feeling that they lacked sufficient time to accurately document patient information or take notes during handovers. This time pressure could lead to errors due to incomplete information being recorded. While some students showed interest in utilising digital recorders for handovers, they recognised that this approach could compromise patient confidentiality (Crouch 2019; King 2019; Ridley 2011). Additionally, students highlighted the necessity for effective strategies to manage their dyslexia within clinical settings. Documentation errors were often associated with time constraints and the inaccurate recording of patient information. Although the use of digital recorders was considered a potential solution, it was largely perceived as a violation of patient confidentiality (Crouch 2019; King 2019; Ridley 2011).

Documentation was another area of concern regarding patient safety. Students shared that they frequently felt they did not have enough time to accurately record patient information or take notes during handovers. This lack of time could result in mistakes due to partial information being documented. Although some students expressed interest in using digital recorders for handovers, they acknowledged that this could breach patient confidentiality (Crouch 2019; King 2019; Ridley 2011). Students expressed the need for effective strategies to manage their dyslexia in clinical settings. Documentation errors were often linked to time constraints and a lack of accurate patient information recording. While the idea of using digital recorders for handovers was considered a potential solution, it was viewed as a violation of patient confidentiality (Crouch 2019; King 2019; Ridley 2011).

Moreover, Morris and Turnbull (2006) emphasised the necessity of allocating more time for patient care, specifically linking it to safety concerns in medication administration. Sixteen out of the 18 students recognised the potential for unsafe practices. They highlighted that having adequate time to concentrate on documentation after patient interventions was crucial. One participant who made an error shared, ‘I was devastated. I didn't think I could carry on. How can someone with dyslexia be a nurse and be safe with patients?’ This comment revealed the emotional burden experienced by students with dyslexia, who felt a significant responsibility to practice safely, especially during busy periods on the ward.

In the study by Child and Langford (2011), most students reported a need for greater confidence in medication calculations, administration and documentation. Some medications were challenging to pronounce and could lead to mistakes, especially when two sounded similar. Misinformation could also occur when describing medications to patients and explaining dosages. Both students with dyslexia and those without dyslexia acknowledged their professional duty to ensure patient safety, as mandated by the NMC Code of Practice (NMC 2018).

Morris and Turnbull (2006) highlighted the essential requirement for students to have sufficient time to focus on documentation following any patient intervention. Concerns about efficiency in medication administration were also raised, with students needing more time to confidently complete medication rounds. One participant voiced the emotional burden of making an error, feeling a heavy responsibility to ensure safe practice, particularly during busy ward conditions.

Child and Langford (2011) noted that both students with and without dyslexia adapted their working methods to mitigate potential barriers in providing safe care. This included developing compensatory coping strategies aimed at enhancing efficiency and productivity in clinical practice.

### 4.10 Compensatory Coping Strategies

Student nurses with dyslexia use various compensatory strategies to successfully navigate their clinical placements. Each student may adopt a unique combination of techniques based on personal preferences and needs, making clinical practice more accessible and fulfilling (King 2019; Crouch 2019; Child and Langford 2011; Ridley 2011; Morris and Turnbull 2006).

Crouch (2019) examined the coping strategies students develop to manage the challenges associated with dyslexia. These strategies include time management techniques and seeking support from peers or educators. Students reported being safety conscious by remaining vigilant and double‐checking their work before administering medications. Some students avoided complex or unfamiliar tasks while on placement, although this was usually only feasible during shorter placements where such avoidance might go unnoticed. A limitation of Crouch's (2019) study was the potential bias, as the researcher was known to the students.

Equally, Ridley (2011) found that students developed coping strategies by cultivating self‐awareness, employing creative problem‐solving skills and engaging in further reading to enhance their knowledge in preparation for their placements. Students felt confident in their verbal communication skills and excelled in practical nursing tasks. Students frequently requested that their practice supervisors double‐check their medication calculations prior to administration.

In Morris and Turnbull's (2006) study, students reported using notepads to take notes, which helped them remember important details during their shifts, especially when clarification was needed. Some students avoided activities that relied heavily on memory recall. One student mentioned hiding in the bathroom until their practice supervisor returned, while another walked away from the phone when it rang. Although these may not be effective coping strategies, students recognised that they needed to address these issues in the future.

A helpful strategy identified was asking for capital letters to be used on drug charts and patient documentation, as this made the information easier to read. However, this often depended on the students' confidence in making such requests. Similarly, asking for additional time to complete paperwork required self‐advocacy. Some students requested a quiet room with minimal distractions, although this was not always attainable.

When considering potential nursing careers, only two out of 18 students in Morris and Turnbull's (2006) study expressed interest in working in acute hospitals, while the other 16 preferred a slower‐paced work environment. This preference arises from the belief that they may struggle to maintain long‐term compensatory strategies in a busy ward. Quieter settings may allow students with dyslexia to develop at their own pace.

### 4.11 Support for Practice Assessors and Supervisors

Morris and Turnbull (2006) highlighted issues related to the support provided by practice assessors. Six students sought assistance but indicated that their assessors or supervisors either needed to improve their support skills or were uninterested in helping. One student refrained from asking for help after overhearing their practice assessor speak negatively about another student with dyslexia, implying that the student was lazy. This underscores the need to raise awareness of dyslexia among staff in clinical settings.

In a follow‐up study, Morris and Turnbull (2007) found that some students had positive support experiences. One student noted that their practice assessor was ‘really understanding’ and dedicated significant time to helping them with care plans. Another student, who had not disclosed their dyslexia, reported that their supervisor observed their slower pace and made necessary allowances. However, two students expressed negative experiences, with their assessors stating they would never succeed as nurses, which resulted in diminished self‐confidence and adversely affected their learning experiences.

King (2019) primarily revealed positive support experiences for students who disclosed their dyslexia. Students reported that their practice assessors and supervisors were very supportive, making reasonable adjustments such as allowing more time for paperwork, helping them practise the pronunciations of medications, and double‐checking their medication dosages. Conversely, one student noted that their practice assessor discouraged them from engaging in certain activities for fear of making mistakes, which raised concerns about accountability.

Ridley (2011) acknowledged that while students appreciated support from their practice assessors or supervisors in clinical practice, not all provided the necessary assistance. One student expressed surprise at the negative perceptions of dyslexia in the nursing environment, especially since nursing is a caring profession. These negative attitudes from practice assessors contradicted the essence of nursing identity. A lack of understanding of dyslexia impacted the support that students received, highlighting the need for change.

Child and Langford (2011) conducted a comparative study of student nurses with and without dyslexia, emphasising the importance of supportive practice assessors and supervisors for both groups. However, there was variability in the perceived quality of the support provided. Students felt there was a need for a greater understanding of the requirements and expectations for student nurses. Additionally, paperwork was often signed off without adequately assessing competency/proficiencies. Most students believed their practice assessors or supervisors did not allocate enough time for their development, negatively affecting their confidence in completing paperwork as part of their Nursing and Midwifery Council (NMC) requirements. This highlights the need for advocacy and support.

The role of practice assessors and supervisors is crucial in supporting students. Practical support positively influences the learning experiences of all students, including those with dyslexia. Patience, encouragement and understanding are essential components that contribute to the success of students.

## 5 Discussion

The findings from Morris and Turnbull (2007), Child and Langford (2011), Ridley (2011) and King (2019) suggest that the disclosure of dyslexia is difficult and stressful, and those who do not disclose may not be able to conceal their disability given the nursing requirements. The Royal College of Nursing (RCN 2023a, 2023b) acknowledges that many students with dyslexia may fear disclosing their disability because of the lack of knowledge among placement staff, challenges in obtaining reasonable adjustments and perceived stigma from nursing staff and student peers. However, RCN promotes inclusivity and diversity, claiming that students with protected characteristics such as dyslexia bring increased resilience, resourcefulness and valuable lived experiences that others can learn from in the workplace (RCN 2023a, 2023b). Openness and support are crucial for creating an inclusive and empowering environment for student nurses with learning difficulties or disabilities (RCN 2023a, 2023b).

In the UK, the Equality Act 2010 stipulates that a person is not legally obliged to tell their employer they have a disability. However, an employer may have a defence against a claim of discrimination if they are genuinely unaware of someone's disability. This reinforces the need for student nurses to disclose their dyslexia regardless of its difficulty.

A study by Major and Tetley (2019) aimed to investigate the experiences of 14 registered nurses with dyslexia in clinical practice by conducting a narrative life course approach. In‐depth interviews revealed that even qualified nurses with dyslexia found it stressful and sometimes intimidating to disclose their dyslexia. Furthermore, Nurses gained the confidence to disclose their dyslexia when they were in supportive environments or worked with supportive staff (Major and Tetley 2019). Unfortunately, the study did reveal that there was still a stigma attached to dyslexia, which may impact student nurses disclosing their dyslexia once qualified.

Furthermore, studies in France (Macaire et al. 2023) and New Zealand (McDonald 2022) recognised that nursing staff's non‐disclosure of dyslexia was concerning. The number of nurses with dyslexia was unclear and considered underreported. Nurses often withheld information out of fear of the consequences, which included stigma and discrimination from coworkers and employers (Macaire et al. 2023; McDonald 2022).

It is possible that nurses are reluctant to admit they have dyslexia because of previous negative experiences at work and school. Once more, nurses with dyslexia worry about being called names like ‘slow’, ‘stupid’ or ‘high risk’ by co‐workers, employers or the general public (Major and Tetley 2019). This shows that disclosure of dyslexia remains an issue not only in the UK but also outside the UK, and more needs to be done to promote a diverse and accepting workforce (Macaire et al. 2023; McDonald 2022).

Registered nurses with dyslexia who are part of the workforce felt they were better practice supervisors and assessors to all students on placement as they were aware of the importance of support and understanding (Major and Tetley 2019). Therefore, it may be beneficial if registered nurses with dyslexia could support students with dyslexia. However, this may impact patient safety if registered nurses and students lack confidence, often leading to feelings of inadequacy, low self‐efficacy and a perception of being less intelligent, which could ultimately affect safe care (Lincoln et al. 2014).

It is morally and ethically important to protect patient safety, ensuring this is always maintained (World Health Organisation 2023). It is suggested that 1 in 10 patients are harmed in healthcare, with more than 50% of incidents being preventable: with medication errors contributing significantly to this statistic (World Health Organisation 2023). However, there appears to be no research showing how many errors have been made by nurses with dyslexia. Drug calculations and administration are critical nursing skills where the occurrence of any error during patient care could lead to harm or possible death (NMC 2018). The findings from the literature review suggest that student nurses with dyslexia lack confidence in drug calculations, administrations, and pronunciations of medications. Reading prescriptions and documentation of medications also proved complex and challenging, highlighting the need for practice assessors and supervisors to be patient with students (King 2019; Crouch 2019; Child and Langford 2011; Ridley 2011). There is no research identifying if patient safety is compromised as a direct result of dyslexia (Morris and Turnbull 2006, 2007; Crouch 2019; King 2019; Child and Langford 2011; and Ridley 2011).

In the UK, the NMC (2018) aims to prioritise patient safety by ensuring robust support, supervision and assessment are provided for student nurses in clinical practice. However, some studies have revealed that not all students with dyslexia feel that they have received adequate support (Morris and Turnbull 2006; Child and Langford 2011; Crouch 2019; and Ridley 2011). All student nurses must abide by the standards of proficiency and the NMC code, based on four themes: prioritising people, preserving safety, practising effectively and promoting professionalism and trust (NMC 2018). Adhering to these standards will allow student nurses with dyslexia to contribute to safe and effective care delivery by achieving structured proficiency and programme outcomes that ensure fitness to practise. It is important to note that if a student with dyslexia who has reasonable adjustments in place fails to meet the minimum fitness to practise criteria, they will not be able to achieve registered nurse status (RCN 2023a, 2023b). Most students with dyslexia can and do complete their course and go on to be employed in the healthcare sector (RCN 2023a, 2023b). 40% of those with dyslexia receive a degree classification of 2.1, although this could still be increased further if students receive the right support (Philion et al. 2021).

The literature review suggests that students with dyslexia know their limitations and use compensatory coping strategies to overcome specific, individualised barriers. Some strategies include seeking support from practice assessors, supervisors, and peers, which involves being confident enough to ask for help and acknowledge a weakness, which can be embarrassing and difficult. Most students play to their strengths, such as their interpersonal skills, and avoid complicated activities (Crouch 2019; Ridley 2011; Morris and Turnbull 2006). Major and Tetley's (2019) study of qualified nurses with dyslexia suggested that nurses grow in confidence and improve their coping strategies when they have gained experience as registered nurses. The compensatory coping strategies used by student nurses with dyslexia appear to continue once qualified. Registered nurses report using notebooks, spell‐checkers, visual aids, templates and taking more time. All nurses discussed that their career choice was influenced by their dyslexia, stating that quieter and less busy wards were desirable (Major and Tetley 2019).

Morris and Turnbull (2006) found that students had similar feelings towards career choices, stating that busy and noisy wards would be challenging to work in once qualified. Findings by King (2019), Crouch (2019), Child and Langford (2011), Ridley (2011), and Morris and Turnbull (2006) revealed that students with dyslexia may face similar challenges but may use different coping strategies. Reasonable adjustments, therefore, require a personalised assessment of each student's needs, in which practice assessors and supervisors can play a vital role.

In clinical placements, students who receive appropriate support can overcome challenges and perform at their best, promoting patient safety. Students may lose confidence if they feel they are not supported in practice, which can have a negative impact on subsequent placements and may result in their unwillingness to disclose their dyslexia. Practice supervisors and assessors can create a more inclusive and conducive learning environment for students if they accommodate reasonable adjustments in line with the legal considerations of the Equality Act 2010. Research findings from Morris and Turnbull (2006), Crouch (2019), Child and Langford (2011) and Ridley (2011) showed that students with dyslexia did not always feel supported in clinical placements, with practice assessors and supervisors' lack of knowledge about dyslexia being a prominent factor.

Furthermore, studies by Crouch (2019) and King (2019) revealed that lecturers, practice assessors and supervisors admitted that they did not know enough about dyslexia and consequently found it challenging to provide appropriate, reasonable adjustments. The NMC (2018) recognises nurses' crucial role in supporting students during their clinical placements and, therefore, implements teaching proficiencies in its Standards for Nurses, Midwives and Nurse Associates (NMC 2018). The NMC Code suggests that qualified nurses provide compelling learning experiences to students by providing supervision, guidance and informative feedback. Students can then integrate theoretical knowledge into practical skills and begin to foster personal development (King 2019). To do this effectively, nurses need to be aware of learning difficulties such as dyslexia and make reasonable adjustments and teaching styles to fully support and teach their students in line with the NMC Code (NMC 2018).

**FIGURE 2:**
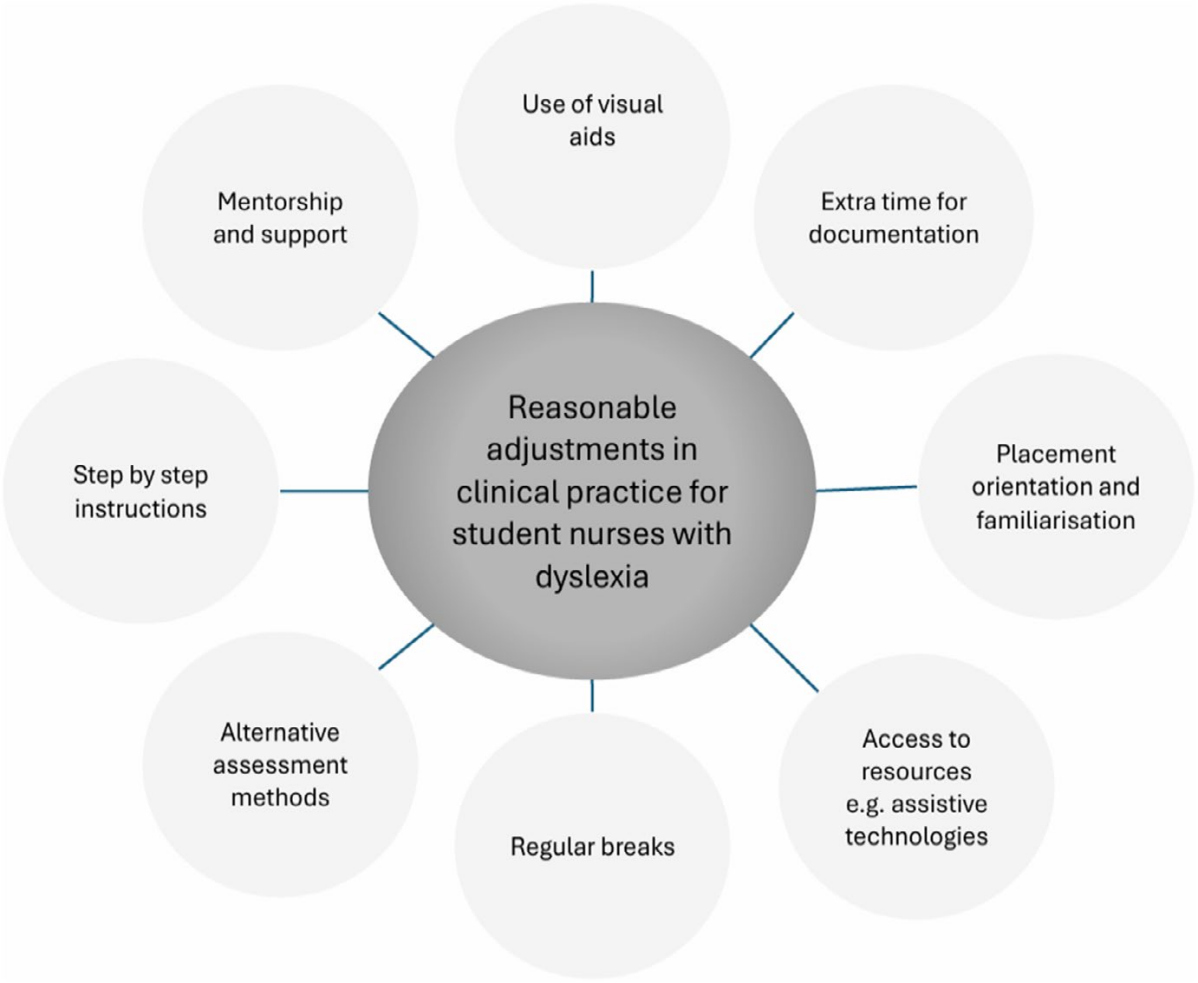
Reasonable adjustments.

In clinical practice, reasonable adjustments for student nurses with dyslexia include allowing extra time for documentation, providing reading materials ahead of time and allowing flexibility in work hours (Walsh's 2021). To supplement oral instructions and explanations, it is also helpful to include a dictionary of terms commonly used on the placement as well as practical demonstrations. Walsh (2021) urges nurses to seek guidance and assistance from the educational institution if they feel a student may be dyslexic so that support can be put in place. Acceptable reasonable adjustments that students with dyslexia could request while in clinical practice are shown in Figure 2.

Factors to be considered when deciding if an adjustment is reasonable include: ‘practicality, effectiveness, efficiency, cost and health and safety (of the individual and others)’ (RCN, 2017). However, there is no duty to make reasonable adjustments that would compromise competence standards. A student with a disability or learning difficulty must be able to demonstrate their fitness to practise, using reasonable adjustments that do not invalidate competence standards (NMC 2018).

**FIGURE 3:**
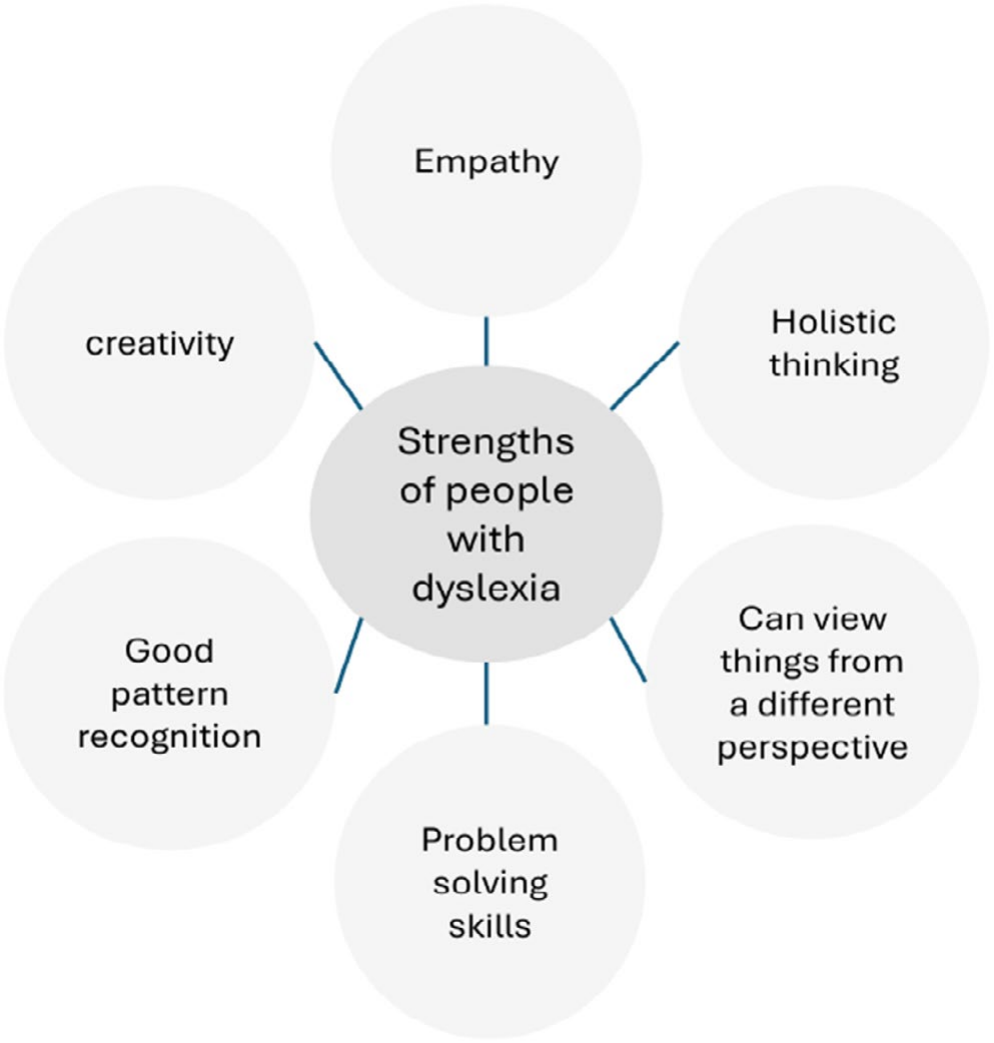
Strengths of dyslexic people in healthcare settings. (Adapted from RCN 2024a, 2024b).

The Royal College of Nursing has developed a neurodiversity guidance toolkit for line managers working in healthcare (RCN 2024a, 2024b) explaining what neurodiversity is, such as dyslexia, dyspraxia and autism. The guidance sets out the benefits of a diverse workplace (Figure 3), the line managers' responsibilities, reasonable adjustments and employers' responsibilities, linking to the Equality Act 2010 to enable line managers to fully support their staff and adopt an inclusive workforce (RCN 2024a, 2024b). However, this guidance is still very new (2024), so no evidence is available to show its effectiveness.

By recognising and valuing the strengths of individuals with dyslexia, the healthcare sector can create a more inclusive and supportive environment that benefits both professionals and patients. Encouraging and supporting student nurses with dyslexia not only helps them achieve their full potential but also enriches the healthcare profession with their unique talents and perspectives (RCN 2024a, 2024b). Fostering a culture of understanding and accommodation, as mandated by the Equality Act 2010, is crucial in ensuring that all healthcare professionals can contribute effectively to patient care and the advancement of the nursing field.

## 6 Conclusion

This review delves into the experiences of student nurses with dyslexia in clinical practice in the UK, highlighting the challenges they face and their coping strategies. Disclosure of dyslexia has been beneficial in obtaining support but is often fraught with complexities and fear of discrimination. The Equality Act 2010 allows for reasonable adjustments to help students achieve their full nursing potential. While medication calculations, administration, and documentation challenges could raise patient safety concerns, student nurses with dyslexia strive to ensure safe and effective care. Understanding these experiences is crucial for enhancing nursing education and creating an inclusive and supportive environment.

## 7 Relevance to Clinical Practice

Exploring the experiences of student nurses with dyslexia enables a better understanding of their unique needs, facilitating the development of targeted support to help them thrive as students and registered nurses. Addressing challenges proactively is essential for maintaining high standards of patient safety. Improved training and support in clinical settings are necessary to raise awareness and understanding of dyslexia.

## 8 Limitations of the Study

The six research papers included in this literature review were all conducted within England and were qualitative studies, which may limit the transferability of the findings to other regions within the UK. Additionally, the structure of nursing degree courses and placements varies across the UK, potentially leading to different experiences for student nurses with dyslexia.

## Conflicts of Interest

The authors declare no conflicts of interest.

## Data Availability

The data that supports the findings of this study are available in the Supporting Information of this article.
